# The power of microRNA regulation—insights into immunity and metabolism

**DOI:** 10.1002/1873-3468.70039

**Published:** 2025-04-11

**Authors:** Stefania Oliveto, Nicola Manfrini, Stefano Biffo

**Affiliations:** ^1^ INGM, National Institute of Molecular Genetics “Romeo ed Enrica Invernizzi” Milan Italy; ^2^ Department of Biosciences University of Milan Italy

**Keywords:** immunity, immunometabolism, metabolism, microRNA, T cells, Treg

## Abstract

MicroRNAs (miRNAs) are a prominent class of small non‐coding RNAs that control gene expression. This comprehensive review explores the intricate roles of miRNAs in metabolism and immunity, as well as the emerging field of immunometabolism. The core of this work delves into the functional and regulatory capabilities of miRNAs, examining their complex influence on glucose and lipid metabolism, as well as their pivotal roles in shaping T‐cell development and function. Specifically, this review addresses how miRNAs orchestrate the complex interaction between cellular metabolic processes and immune responses, underscoring the essential nature of these small regulatory molecules in maintaining homeostasis. Finally, we examine the emerging role of Artificial Intelligence (AI) in miRNA research, focusing on how machine learning techniques are revolutionizing the identification and validation of potential miRNA biomarkers. By integrating these diverse aspects, this review underscores the multifaceted roles of miRNAs in biological processes and their significant potential in advancing biomedical research and clinical applications.

## Graphical abstract

MicroRNAs are emerging as crucial regulators at the intersection of metabolism and immunity. This review examines how miRNAs coordinate glucose and lipid metabolism while simultaneously modulating T‐cell development and immune responses. Moreover, it highlights how cutting‐edge artificial intelligence applications can identify miRNA biomarkers, offering new perspectives for disease diagnosis and treatment.

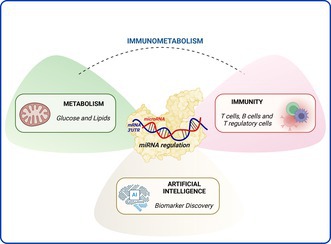

## Abbreviations

**ABCA1**, ATP‐binding cassette subfamily A member 1

**ACC1**, acetyl‐CoA Carboxylase 1

**AI**, artificial Intelligence

**AID**, activation‐Induced Cytidine Deaminase

**AMPK**, AMP‐activated protein kinase

**ANN**, artificial Neural Network

**APOB**, apolipoprotein B

**ATP**, adenosine Triphosphate

**BCL11B**, B‐Cell CLL/Lymphoma 11B

**BCR**, B‐cell receptor

**BIM**, BCL2‐interacting mediator of cell death

**BLIMP‐1**, B lymphocyte‐induced maturation protein‐1

**BTK**, Bruton's tyrosine kinase

**cAMP**, cyclic adenosine monophosphate

**Cdc25a**, cell division cycle 25A

**CLPs**, common lymphoid progenitors

**CNN**, convolutional neural network

**CPT1A**, carnitine palmitoyltransferase 1A

**CROT**, carnitine O‐octanoyltransferase

**CTLA‐4**, cytotoxic T‐lymphocyte‐associated protein 4

**CTLs**, cytotoxic T lymphocytes

**CXCXR5**, C‐X‐C chemokine receptor type 5

**DL**, deep learning

**DN**, double‐negative

**DP**, double‐positive

**DUSP5**, dual specificity phosphatase 5

**DUSP6**, dual specificity phosphatase 6

**EOMES**, eomesodermin

**ERLIN2**, endoplasmic reticulum lipid raft‐associated 2

**ESCC**, esophageal squamous cell carcinoma

**ETS1**, ETS proto‐oncogene 1

**FASN**, fatty acid synthase

**FOXA1**, Forkhead Box A1

**FOXO1**, Forkhead Box O1

**FOXP1**, Forkhead Box P1

**FOXP3**, Forkhead Box P3

**GARP**, glycoprotein A repetitions predominant

**GCK**, glucokinase

**GLP‐1**, glucagon‐like peptide‐1

**GLUT1**, glucose transporter 1

**HADHB**, hydroxyacyl‐CoA dehydrogenase trifunctional multienzyme complex subunit beta

**HDL**, high‐density lipoprotein

**HFN4A**, hepatocyte nuclear factor 4 alpha

**HIF‐1α**, hypoxia‐inducible factor 1‐alpha

**HK2**, hexokinase 2

**HMGCR**, 3‐hydroxy‐3‐methylglutaryl‐CoA reductase

**IFN‐γ**, interferon gamma

**IFN‐γRα**, interferon gamma receptor alpha

**IL‐10**, interleukin‐10

**IL‐10R**, interleukin‐10 receptor

**IL‐2**, interleukin‐2

**IL2RB**, interleukin‐2 receptor subunit beta

**IL‐4**, interleukin‐4

**IL‐7**, interleukin‐7

**IRAK1**, interleukin‐1 receptor‐associated kinase 1

**IRF4**, interferon regulatory factor 4

**LASSO**, least absolute shrinkage and selection operator

**LDHA**, lactate dehydrogenase A

**LDL**, low‐density lipoprotein

**LDLR**, low‐density lipoprotein receptor

**LKB1**, liver kinase B1

**LXR**, liver X receptor

**MCAT**, carnitine O‐octanoyltransferase

**miRNAs**, microRNAs

**ML**, machine learning

**mRNA**, messenger RNA

**Mtb**, Mycobacterium tuberculosis

**mTOR**, mammalian target of rapamycin

**MTPN**, myotrophin

**NADH**, nicotinamide adenine dinucleotide

**NAFLD**, non‐alcoholic fatty liver disease

**ORF**, open reading frame

**OXPHOS**, oxidative phosphorylation

**PCSK9**, proprotein convertase subtilisin/kexin type 9

**PDCD4**, programmed cell death 4

**PDHA1**, pyruvate dehydrogenase e1 subunit alpha 1

**PDK1**, phosphoinositide‐dependent protein kinase‐1

**PD‐L1**, PROGRAMMED DEATH‐LIGAND 1

**PI3K**, phosphoinositide 3‐kinase

**PK**, pyruvate kinase

**PPARs**, peroxisome proliferator‐activated receptors

**PRDM1**, PR domain zinc finger protein 1

**PTEN**, phosphatase and tensin homolog

**RCT**, reverse cholesterol transport

**RISC**, RNA‐induced silencing complex

**RNA**, ribonucleic acid

**RNN**, recurrent neural network

**RORγ**, RAR‐related orphan receptor gamma

**S1P1**, sphingosine‐1‐phosphate receptor 1

**SCD1**, stearoyl‐CoA desaturase‐1

**SHIP‐1**, SH2‐containing inositol phosphatase‐1

**SIRT6**, sirtuin 6

**SOCS1**, suppressor of cytokine signaling 1

**SOCS3**, suppressor of cytokine signaling 3

**SP1**, specificity protein 1

**SREBPs**, sterol regulatory element‐binding proteins

**STAT1**, signal transducer and activator of transcription 1

**STAT3**, signal transducer and activator of transcription 3

**STAT5**, signal transducer and activator of transcription 5

**SVM**, support vector machine

**T2D**, type 2 diabetes

**T‐bet**, T‐box transcription factor TBX21

**TCR**, T cell receptor

**Tfh**, T follicular helper

**TFs**, transcription factors

**TGF‐β**, transforming growth factor beta

**Th1**, T helper 1

**Th17**, T helper 17

**Th2**, T helper 2

**TLR**, toll‐like receptor

**Tr1**, type 1 regulatory T cells

**TRAF6**, TNF receptor‐associated factor 6

**Tregs**, regulatory T cells

**UTR**, untranslated region

**VEGFA**, vascular endothelial growth factor A

MicroRNAs (miRNAs) represent one of biology's most compelling regulatory mechanisms. They are small non‐coding RNAs (ranging from 19 to 25 nts in length) which negatively modulate gene expression at the mRNA and protein level. By annealing to coding transcripts, miRNAs cause their translation inhibition and degradation [1]. miRNA genes progress through multiple processing stages, in which they are processed into pre‐miRNA and finally into mature miRNA [2]. Mature miRNAs exhibit tissue‐specific expression patterns that may result in tissue‐specific effects [3, 4].

**Fig. 1:**
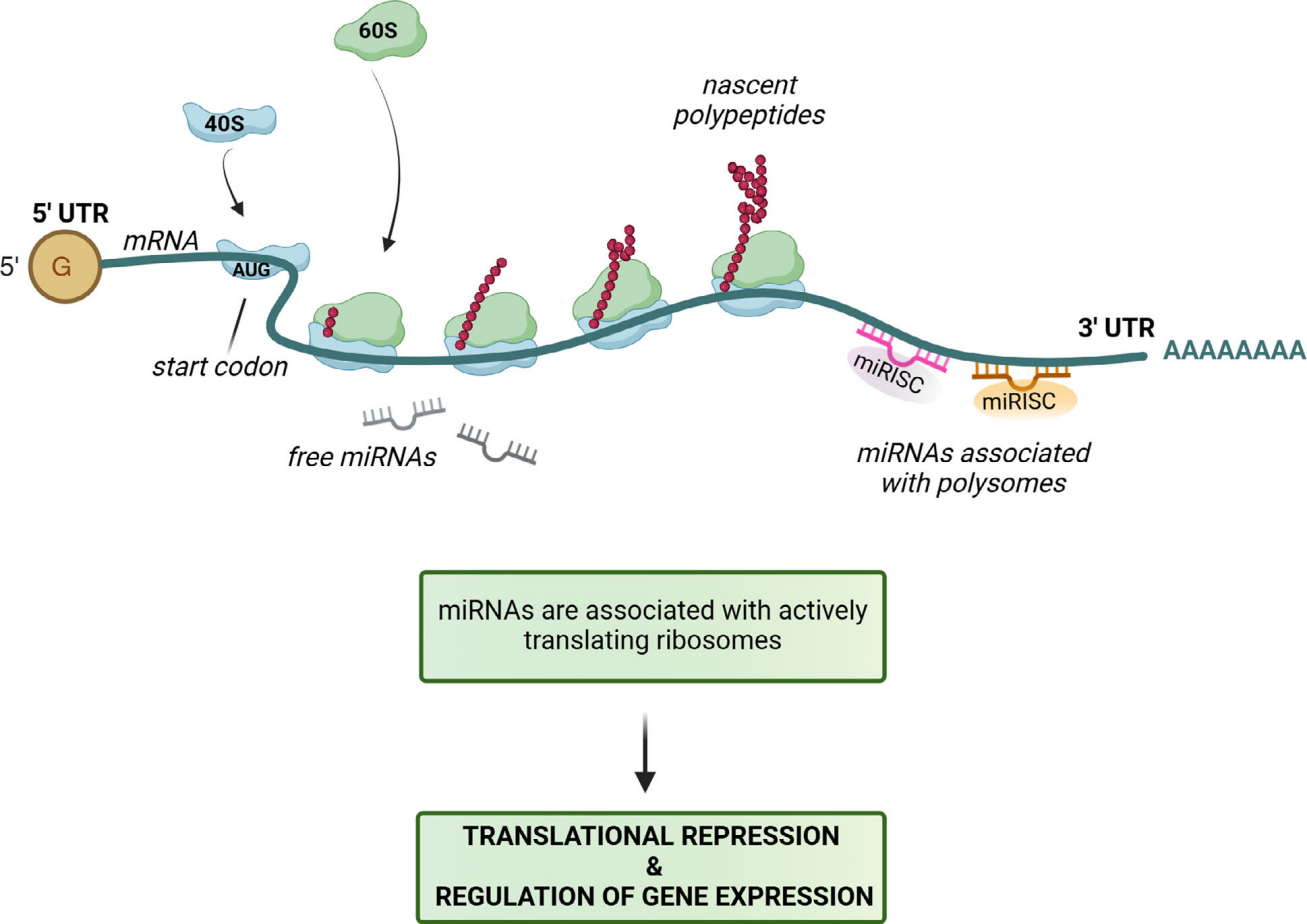
Association of miRNAs with the translational machinery. Representation of the relationship between miRNAs and the translational machinery. An mRNA molecule with its key structural elements is shown. The figure depicts two populations of miRNAs. Free miRNAs are shown floating in the cytoplasm, while another set of miRNAs is illustrated in direct association with the polysomes. This association is particularly evident near the 3′ UTR of the mRNA, a common target site for miRNA binding. The miRNA‐induced silencing complex (miRISC) is shown bound to the 3′ UTR, emphasizing the site of miRNA action. The bottom of the figure emphasizes that the interaction between miRNAs and actively translating ribosomes leads to downstream effects on protein production and overall gene expression.

Originally discovered over two decades ago, miRNAs have emerged as master regulators of gene expression, capable of fine‐tuning the expression of hundreds of genes simultaneously through base‐pairing interactions [5, 6]. miRNAs predominantly bind to the 3′ untranslated region (3′UTR) of messenger RNAs (mRNAs) and are enriched on actively translating ribosomes [7] (see Box 1 for details and Fig. 1). The mechanism of miRNA‐mediated gene silencing was proposed to follow a sequential pattern. Firstly, miRNAs repress translation of their target mRNAs, an event followed by the coordinated removal of two key transcript protection features: the poly(A) tail, through deadenylation, and the 5′ cap structure, through decapping. These modifications leave the mRNA exposed and susceptible to degradation by cellular exonucleases [8, 9, 10]. This mechanism is, however, still debated since some studies suggest that destabilizing target mRNAs is the primary mechanism for reducing protein output [11, 12]. We detail this issue in Box 1.

Box 1 **miRNAs and translation.**

The framework by which miRNAs regulate translation is a pillar of biochemistry. We have discussed in detail these aspects in a previous review [1]. Hereafter, we will summarize the essential steps and will integrate them with novel knowledge. Translation is conventionally divided into three phases: initiation, elongation and termination [13]. At initiation, a single mRNA is recruited onto ribosomes by initiation factors. Given that initiation is the rate limiting step of protein synthesis [14] it is also the step involved in the mechanism of miRNA inhibition. Given that miRNAs in the RISC complex bind seed sequences in the 3′ UTR of target mRNAs inducing their deadenylation and degradation [15], the critical question of whether translational repression is required for mRNA degradation or whether translational repression can occur without mRNA degradation arises. In general, the current view is that the balance between miRNA‐induced mRNA decay and translational repression is rather intricate [10]. Mechanistically, the most relevant observations are described here: mRNAs have a regulatory 5′ UTR [16] recognized by initiation factors, a start codon upstream of the Open Reading Frame (ORF), a stop codon downstream of the ORF and a 3′ UTR that controls localization and stability of the mRNA [17]. The average length of the 3′ UTR, downstream of a stop codon, is around 350 nucleotides [18]. It is rationalized that miRNAs stably bind the 3′ UTR of mRNAs. The preferential binding to the 3′ UTR is due to the fact that the 5′ UTR and the ORF, even if they contain target sequences, are populated by scanning and elongating ribosomes, respectively, that impede correct sequence recognition. However, reports on the presence of active miRNAs on the 5′ UTR or ORF exist [19]. Concerning the 5′ UTR, seminal work has reported that multiple miRNA seeds in the 5′ UTR contribute to translational downregulation through their capability to destabilize mRNAs [20]. However, the prevalent modality of miRNA action occurs through binding to the 3′ UTR. Occasionally, miRNAs can reduce the translation of specific mRNAs in the absence of detectable mRNA degradation, although it should be noted that most studies have analyzed steady‐state levels of mRNAs, thus fully neglecting the contribution of new transcription. What is clear is that translational downregulation seems a prerequisite for target degradation [21]. The eIF4F complex binds the 5′ cap of mRNA, and this interaction is essential for miRNA‐mediated translational downregulation and mRNA degradation [22, 23]. In addition, evidence has shown that active miRNAs are highly enriched on translating ribosomes [7]. For details and alternative mechanisms refer to specialized reviews such as [1, 24].

The precise sequence of miRNA‐driven mRNA translational inhibition and degradation remains an unresolved question in current research. Several years ago a work directed by Guo *et al*. showed that miRNA‐induced changes in mRNA levels closely reflect gene expression impacts, with mRNA destabilization being the primary mechanism for reduced protein output. [11]. Kinetic analysis has shown that miRNAs primarily repress translation of new targets, followed by mRNA deadenylation and decay, clarifying their silencing mechanism [8, 25]. Interestingly, there is also another hypothesis: miRNAs destabilize mRNAs when not ribosome‐associated, but, at the same time, the miRNAs that target mRNAs remain fully engaged with polysomes. The study demonstrated that while the mRNAs are polysome‐associated, the decapping mechanism progresses from 5′ to 3′ direction, following the last translating ribosome [1, 26]. According to this model, miRNA‐mediated mRNA decay occurs co‐translationally, providing a potential solution to the complex question regarding the relationship between miRNA‐driven translational inhibition and mRNA degradation. The idea is that these processes are intricately linked and happen concurrently. In conclusion, the most relevant route for inhibition by miRNAs is binding to the 3′ UTR of mRNAs, preferentially on mRNAs engaged by the translational machinery. This fact makes sense because targeting of translationally silent mRNAs would not lead to obvious physiological effects. Interestingly, downregulation of expression obtained by miRNA targeting normally ranges in the order of 50–70%, making this mechanism compatible with modulation of essential genes.

miRNAs do not exist in a free form. They are always loaded onto the RISC complex that confers upon them the ability to silence their target transcripts through base‐pairing interactions. At the core of miRNA function is the *seed sequence*, located at nucleotides 2–8 from the 5′ end of the mature miRNA. The seed sequence plays a pivotal role in target recognition and specificity and is primarily responsible for miRNA‐mRNA interactions [5, 6]. This plasticity is further enhanced by the existence of isomiRs (mature miRNA variants that retain the seed sequence but may differ in length and/or sequence) [27, 28] and RNA editing, an essential epitranscriptional modification of miRNA nucleotide sequences, potentially modifying their biogenesis and target‐binding capabilities [29]. The biological significance of both isomiRs and sequence editing is becoming increasingly recognized, as they add an extra layer of complexity to miRNA‐mediated gene regulation and expand or alter the regulatory repertoire of their parent miRNAs.

Last, but not least, the functional spectrum of miRNAs extends well beyond simple gene silencing. Their regulatory capacity depends on both their own expression levels and those of their target mRNAs [30] and is also influenced by their genomic location, which is either in intragenic or intergenic regions [31] (Box 1). Moreover, many miRNAs exhibit species‐ and/or tissue‐specific expression patterns, necessitating co‐localization with their targets for functionality [6, 7].

The profound impact of miRNAs on gene regulation, coupled with their tissue‐specificity and stability in biological fluids, has positioned them as promising biomarkers. It is important to underline that while miRNA research often examines individual miRNA‐target interactions, contemporary understanding emphasizes the network‐based nature of miRNA function. Most miRNAs simultaneously regulate multiple target genes, often within the same biological pathway or process, creating complex regulatory networks rather than simple linear relationships. We discuss specific miRNA‐target interactions that have been experimentally validated and provide valuable insights into miRNA mechanisms. However, these should be interpreted within the broader context of miRNA regulatory networks, where the full functional significance of miRNAs can only be appreciated when considering their integrated effects on multiple targets within specific cellular contexts. This balanced perspective allows us to learn from focused mechanistic studies while acknowledging that isolated interactions rarely capture the complete biological impact of a miRNA.

Here, we explore miRNA regulatory mechanisms in metabolism and their impact in modulating immune responses, encompassing adaptive immunity and the emerging field of immunometabolism. Finally, we discuss the integration of artificial intelligence in miRNA research, highlighting state‐of‐the‐art computational approaches in miRNA discovery, target prediction, and biomarker identification. This synthesis of current knowledge aims to deepen our understanding of the diverse functions of miRNAs and explore their potential clinical application.

## Physiological relevance of miRNAs in metabolism and immunity

miRNAs orchestrate complex biological functions by regulating protein expression across various cellular contexts. Of particular interest is their significant impact on metabolism and immunity, two fundamental and interconnected aspects of organismal regulation. In metabolism, miRNAs act as key modulators of energy homeostasis, influencing processes from glucose metabolism to lipid synthesis. They function as molecular switches, rapidly adjusting metabolic pathways in response to nutritional and environmental cues. In the immune system, miRNAs shape both innate and adaptive responses by regulating the development, differentiation, and function of immune cells. The realms of metabolism and immunity are not isolated but are deeply interconnected, and miRNAs stand at this crossroad, simultaneously regulating metabolic pathways and immune functions. Here we will examine and discuss key miRNAs involved in metabolic and immune processes.

### miRNAs as central regulators of energy metabolism

If we imagine metabolism as a biochemical symphony, it would be a finely tuned performance where each enzyme and substrate plays a fundamental role in transforming dietary nutrients into a complex, energy‐rich repertoire that sustains organismal growth, development, and survival. Within this intricate metabolic orchestra, miRNAs have emerged as central regulatory hubs, integrating and fine‐tuning the expression of key metabolic pathways. A pivotal question that arises is how these small non‐coding RNAs regulate the complex network of metabolic pathways and what the molecular mechanisms are by which miRNAs modulate the activity of critical metabolic enzymes. In this review, we will focus our discussion on the regulatory influence of miRNAs over the metabolism of two primary energy sources: glucose and lipids.

Glucose enters cells through GLUT transporters and, once phosphorylated to glucose‐6‐phosphate, undergoes anaerobic glycolysis to generate pyruvate, producing ATP and NADH. Pyruvate is then converted to acetyl‐CoA by mitochondrial pyruvate dehydrogenase. Acetyl‐CoA then enters the Krebs cycle, generating additional high energy and reducing molecules through aerobic glycolysis. Lipids are also processed in the mitochondria through β‐oxidation. Specifically, fatty acids are firstly activated to fatty acyl‐CoA, then transported into mitochondria and ultimately undergo repeated oxidation cycles. [32]. The interplay between glucose and lipid metabolism is critical for energy homeostasis, with excess glucose being converted to fatty acids for storage, and stored lipids being broken down during fasting to provide energy. This intricate balance is essential for the biosynthesis of macromolecules and for understanding metabolic disorders [33].

### The miRNA‐glucose nexus: Multifaceted interactions in metabolic network

Metabolic cues, including alterations in nutrient, hormone, and cytokine levels, modulate miRNA expression and biogenesis. miRNAs, for their part, influence the cellular response to those same metabolic stimuli, creating a feedback loop where alterations in miRNA levels can induce changes in gene expression [34]. We can define this interplay as a key mechanism that allows cells to adapt their metabolic responses with remarkable speed and precision. Existing research has predominantly focused on the effects of primary nutrients, such as glucose and lipids, on miRNA expression, leaving a significant gap in our understanding of the broader spectrum of dietary influences which affect miRNA‐mediated metabolic control [35, 36, 37]. Glucose homeostasis, a critical metabolic parameter, has been shown to modulate miRNA expression, with high glucose levels upregulating miR‐124a in pancreatic β‐cells, thereby affecting insulin secretion [38]. Conversely, glucose deprivation in cancer cells induces miR‐451 expression, which by targeting members of the LKB1 kinase complex as well as several downstream effectors, negatively regulates the AMPK signaling pathway [39]. Moreover, it has been shown that incubation of freshly isolated rat pancreatic islets or INS‐1E insulinoma cells with glucose reduces miR‐375 levels [40]. Yet, hyperglycemia also upregulates miR‐29c, reducing Sprouty homolog 1 (Spry1) expression and promoting cell apoptosis and fibronectin synthesis in diabetic nephropathy. Similar effects are also observed for other miRNAs [41], suggesting that therapeutic approaches targeting miRNAs must be carefully tailored to specific metabolic states. Lipid intake is also involved in miRNA modulation. High‐fat diets alter hepatic expression of miR‐122, influencing lipid metabolism and potentially contributing to fatty liver disease [42, 43]. Moreover, mice with miR‐378 upregulation in the liver under high‐fat diets show increased susceptibility to obesity, while genetic disruption of miR‐378 provides resistance to diet‐induced weight gain [44, 45]. This is particularly intriguing as it suggests that dietary interventions could potentially be used to modulate miRNA expression and function as a preventive strategy against metabolic disorders. Also, dietary patterns, such as caloric restriction, have been associated with changes in miR‐335 expression, potentially contributing to its effects on longevity and metabolism [36]. Furthermore, dietary fiber intake can modulate host miRNA expression, particularly levels of miR‐375 and miR‐124 in the colon, by influencing gut microbiota‐derived short‐chain fatty acids [46].

Glucose levels in the blood are tightly controlled, and fluctuations from the normal situation are associated with harmful diseases, such as diabetes and metabolic syndrome [35]. Multiple organs play critical roles in glucose homeostasis. The pancreas secretes insulin and glucagon, which reduce and increase blood glucose, respectively; muscles oxidize glucose to produce energy, and fat and liver act as rapid and slow modulators of glucose levels. In particular, insulin promotes glucose uptake in peripheral tissues and inhibits hepatic glucose production, while glucagon stimulates glucose release from the liver. miRNAs acting as attenuators of insulin expression and secretion are well characterized. One way miRNAs regulate insulin expression and secretion is by targeting key enzymes involved in pancreatic β‐cell glucose catabolism and ATP generation [35]. Overexpression of miR‐375 reduces insulin secretion by β‐cells, while its inhibition enhances secretion [47, 48]. miR‐375 plays a critical role in β‐cell function, targeting genes like phosphoinositide‐dependent protein kinase‐1 (PDK1) that are involved in insulin secretion [49]. miR‐130a/b and miR‐152 also exert a significant influence on β‐cell metabolism by targeting two enzymes in the glucose catabolism pathway: (1) glucokinase (GCK), the enzyme catalyzing the first and rate‐limiting step of glycolysis in β‐cells; and (2) Pyruvate Dehydrogenase E1 subunit‐α1 (PDHA1), a key component of the pyruvate dehydrogenase complex that converts pyruvate to acetyl‐CoA in mitochondria. By suppressing these enzymes, miR‐130a/b and miR‐152 effectively decrease the intracellular ATP/ADP ratio. This metabolic alteration consequently leads to diminished insulin synthesis and secretion [50, 51]. More generally, the ATP/ADP ratio is critical for insulin secretion. Insulin expression is stimulated by an elevated ATP/ADP ratio, which in turn controls insulin secretion from β‐ cells [52]. Accordingly, miRNAs that target mitochondrial proteins or enzymes involved in the electron transport chain can significantly affect ATP generation and, consequently, insulin secretion. Among them, miR‐33a and miR‐33b have been found to target key enzymes in the mitochondrial respiratory chain [53, 54], as well as sirtuin 6 (SIRT6) and NAD^+^‐dependent histone deacetylase, which regulate glucose metabolism and stress resistance [55, 56]. Due to their pleiotropicity, some miRNAs directly target insulin gene expression. For instance, miR‐9, which also regulates Onecut2, the insulin gene transcriptional repressor [57, 58] and miR‐204, which directly targets insulin gene expression in pancreatic β‐cells [59]; these data are, however, controversial [60]. In addition, miR‐7 exhibits a dual regulatory role in β‐cell function: beyond its influence on insulin expression, it also modulates insulin secretion by targeting proteins involved in cytoskeletal reorganization. By repressing cytoskeletal components, miR‐7 effectively reduces the quantity of insulin released from β‐cells [61, 62]. Insulin secretion from pancreatic β‐cells is a complex process that culminates in the exocytosis of insulin‐containing granules. Among the miRNAs identified as regulators of this process, miR‐124a has a prominent role. It targets Rab27A [63, 64], a small GTPase essential for the docking of insulin granules to the plasma membrane, thereby impairing formation of the Nucleolar Complex Protein 2 (Noc2) complex, which is involved in the fusion of insulin granules with the plasma membrane [65]. By doing so, miR‐124a influences both the number of insulin granules and the efficiency of their fusion and release. In addition, miR‐9 and miR‐375 function as negative regulators of insulin exocytosis. miR‐9 targets Granuphilin/Slp4, while miR‐375 regulates Myotrophin (MTPN), a protein involved in the final step of insulin granule fusion [58]. Moreover, miR‐26a has also been found to target several genes involved in the exocytosis machinery, directly influencing the efficiency of insulin granule fusion with the plasma membrane [66]. In conclusion, miRNAs are heavily involved in the production and secretion of insulin, the main controller of blood glucose levels. In contrast, the interplay between miRNAs and glucagon signaling remains largely unexplored. This paucity of information may be attributed to either a limited role of miRNAs in regulating glucagon signaling pathways or, more likely, a lack of focused research in this specific area [35, 67, 68]. However, recent studies have shown that miR‐194 suppresses GLP‐1 synthesis by inhibiting the expression of the Wnt signaling effector beta‐catenin and the forkhead box a1 (Foxa1) protein, suggesting a role for miRNAs in the modulation of glucagon‐like peptide signaling with potential implications for the management of obesity and metabolic disorders [69].

In addition to their role in controlling insulin production, miRNAs also regulate the influx and utilization of glucose within cells. GLUT1, a glucose transporter, can be downregulated by miR‐143, which inhibits glucose uptake and glycolysis in T cells [70]. miRNAs can fine‐tune the cell's response to glucose by also regulating glycolytic enzymes, adding a new intricate level of complexity to this mechanism. HK2 is regulated by miR‐125a and miR‐143 [71, 72]. Glucokinase (GCK), the glucose sensor of β‐cells, is targeted by several miRNAs, including miR‐206 [73, 74]. Modulating the cell's ability to phosphorylate and trap glucose affects downstream glycolytic flux. Pyruvate kinase (PK), the enzyme involved in the final step of the glycolytic pathway, is also regulated by miR‐122, thereby affecting the generation of pyruvate for mitochondrial metabolism [75]. LDHA catalyzes the conversion of pyruvate to lactate while simultaneously oxidizing NADH to NAD^+^. This reaction is crucial under conditions in which oxygen is limited, such as in anaerobic metabolism, or in cells that rely heavily on glycolysis for energy production. miR‐34a has been shown to target LDHA, potentially favoring mitochondrial pyruvate oxidation and enhancing glucose‐stimulated insulin secretion [76]. miR‐199a‐3p inhibits LDHA expression by downregulating Sp1, revealing a novel miR‐199a‐3p/Sp1/LDHA axis that critically influences aerobic glycolysis [77]. In conclusion, miRNAs are emerging as critical regulators of glucose metabolism, with multifaceted roles in various aspects of glucose homeostasis, from glucose sensing and uptake, to insulin production, secretion, and functionality. The involvement of miRNAs in glucose metabolism opens up possibilities for developing novel therapeutic strategies for metabolic disorders. However, the complexity of these networks presents significant challenges in translating them into clinical applications.

### miRNAs in lipid metabolism

Lipids are a diverse group of biomolecules that play different roles in energy storage, cellular structure, and signaling pathways. Cholesterol and phospholipids are integral components of cell membranes, while triglycerides function as the primary form of energy storage in adipose tissue. The lipid pool of the cell is maintained through a combination of dietary uptake and *de novo* synthesis, with the liver serving as the primary site for endogenous lipid production.

Cholesterol, fatty acid, and phospholipid biosynthesis are regulated by transcription factors like Sterol Regulatory Element‐Binding Proteins (SREBPs), Liver X Receptors (LXRs), and Peroxisome Proliferator‐Activated Receptors (PPARs). miRNAs have emerged as critical modulators of these lipid homeostasis regulators, adding a layer of control that was largely unknown just a few decades ago.

One example of this intricate miRNA‐mediated regulation is miR‐7, which can indirectly lead to the activation of SREBP1, a key transcription factor involved in lipid homeostasis. This is achieved not through direct activation, as miRNAs typically function as repressors, but through a complex regulatory mechanism [78] through which miR‐7 targets and represses the expression of the Endoplasmic Reticulum Lipid Raft‐Associated 2 protein (ERLIN2), which is involved in the degradation of activated SREBP1. By repressing ERLIN2, miR‐7 indirectly leads to increased levels of active SREBP1, thereby influencing lipid metabolism [79].

The activity of LXRs, key TFs involved in lipid homeostasis, is modulated by miRNAs. Specifically, miR‐613 was shown to target the LXRα gene, affecting cholesterol efflux and fatty acid synthesis [80]. Additionally, the expression of PPARγ, which is essential for adipocyte differentiation and lipid storage, was shown to be regulated by several miRNAs including miR‐27a, which was indeed shown to inhibit adipogenesis [81], and miR‐7, which directly targets the 3′ UTR of PPARγ mRNA, in turn inhibiting adipocyte differentiation and regulating lipid accumulation and expression of adipogenic markers [78]. Interestingly, PPARγ activation *per se* influences miR‐7 expression, suggesting a potential feedback loop [82]. Lastly, miR‐34a represses HNF4A expression, mediating the hepatic response to metabolic stress during lipid overload [35]. The intricate regulation of lipid metabolism extends beyond cellular processes and systemically influences lipid homeostasis. Lipid transport in the circulatory system is mediated by lipoproteins, including Low‐Density Lipoprotein (LDL), and High‐Density Lipoprotein (HDL). LDL particles are synthesized in hepatocytes and primarily function in cholesterol delivery to peripheral tissues. Conversely, HDL facilitates Reverse Cholesterol Transport (RCT), removing excess cholesterol from peripheral tissues and transporting it to the liver for excretion or recycling. The LDL/HDL balance is critical for lipid homeostasis. The miR‐33 family, including miR‐33a and miR‐33b, are located in intronic regions of SREBP2 and SREBP1, respectively, and are the most extensively investigated miRNAs and key modulators of lipid metabolism genes [53, 83]. Co‐transcribed with their respective host genes, both miRNAs regulate comparable physiological processes, creating in this way an intricate regulatory circuit. When cellular sterol levels are low, SREBP2 is activated, leading to increased cholesterol biosynthesis and uptake, while simultaneously expressing miR‐33a. Similarly, when SREBP1 is activated under low sterol conditions, it co‐expresses miR‐33b [84]. In detail, both miRNAs target ABCA1 (ATP‐binding cassette transporter A1), a critical protein in HDL biogenesis and reverse cholesterol transport. By repressing ABCA1, miR‐33a/b reduces cholesterol efflux to apolipoprotein A1, thereby decreasing HDL levels [85, 86]. Lipid synthesis is counterbalanced by fatty acid oxidation. miR‐33a/b target genes are also involved in fatty acid oxidation, including Carnitine O‐Octanoyltransferase (CROT), Carnitine Palmitoyltransferase 1A (CPT1A), and Hydroxyacyl‐CoA Dehydrogenase Trifunctional Multienzyme Complex Subunit Beta (HADHB). This repression leads to decreased fatty acid oxidation and increased cellular lipid accumulation [54, 86, 87]. The physiological importance of miR‐33a/b has been demonstrated in various *in vivo* studies. Anti‐miR‐33 therapy in mice increases hepatic expression of ABCA1 and HDL plasma levels [88]. Moreover, miR‐33 inhibition promotes reverse cholesterol transport and regression of atherosclerotic plaques in mouse models of atherosclerosis [89]. While mice express only miR‐33a, humans express both miR‐33a and miR‐33b [90]. This difference may contribute to species‐specific regulation of lipid metabolism and should be considered when translating findings from mouse models to humans. In addition miR‐33a/b are able to control upstream regulators of fatty acid and lipid homeostasis [56]. miR‐33 targets SIRT6, leading to its downregulation and resulting in enhanced SREBP activity and increased lipogenesis [91]. Interestingly SIRT6 also directly regulates SREBP1 target genes, namely ACC1, SCD1, and FASN, which are relevant for fatty acid production [56]. This regulatory axis highlights a mechanism by which miR‐33 fine‐tunes lipid metabolism by modulating SIRT6 levels. By regulating cholesterol homeostasis and fatty acid oxidation, miR‐33a/b directly affects HDL production and indirectly LDL metabolism.

In this context, several miRNAs regulate LDL metabolism, influencing both its production and clearance. miR‐128 and miR‐148a directly target the LDL Receptor (LDLR) transcript, reducing LDLR protein levels and consequently decreasing LDL uptake [92, 93, 94]. Conversely, miR‐185 enhances LDLR expression by targeting Proprotein Convertase Subtilisin/Kexin type 9 (PCSK9), a negative regulator of LDLR [95, 96]. Moreover, miR‐34a targets apolipoprotein B (APOB), the main protein component of LDL, affecting LDL particle assembly and secretion [97], and miR‐21 targets 3‐Hydroxy‐3‐MethylGlutaryl‐CoA Reductase (HMGCR), the relevant enzyme in cholesterol biosynthesis, indirectly influencing LDL levels [98]. Of particular importance is that certain miRNAs (e.g. miR‐122, miR‐33a) have been found circulating in association with LDL particles, potentially serving as biomarkers for cardiovascular diseases [99, 100]. Other miRNAs involved in the complex process of lipid metabolism are miR‐370 [101] and miR‐758, which post‐transcriptionally modulates the levels of the ABCA1 cholesterol transporter in macrophages, influencing the regulation of cholesterol efflux, a critical process in reverse cholesterol transport [102]. Although the liver is the primary site of lipid metabolism, adipose tissues play an equally important role, particularly for lipid storage and energy homeostasis. The differentiation of white adipocytes, relevant for lipid storage, is regulated by a suite of miRNAs, such as miR‐103/107, miR‐378/378*, miR‐143, miR‐21, miR‐27a, and miR‐130 [103]. These miRNAs regulate adipogenic factors and lipid metabolism genes, controlling adipocyte differentiation and function [34].

### Extracellular and intracellular miRNAs: Bridging the glucose and lipid pathways

The interplay between lipid and glucose metabolism is a critical nexus in cellular energetics, where regulatory pathways intersect to maintain metabolic homeostasis and adapt to varying nutritional states and energy demands. In Table 1, we summarize the key miRNAs involved in glucose and lipid metabolism, highlighting those with dual regulatory roles. By simultaneously modulating components of lipid and glucose metabolism pathways, miRNAs ensure a coordinated cellular response to energy demands and nutritional states. The dual regulatory role of many miRNAs in both lipid and glucose metabolism underscores the interconnected nature of these pathways and the efficiency of miRNA‐mediated regulation. Specifically, the miR‐33 family regulates cholesterol homeostasis and glucose metabolism by targeting components of the insulin signaling pathway [53, 84]. miR‐122, miR‐34a and miR‐143 have a role in both lipid metabolism and glucose homeostasis [104, 105]. miRNAs function not only as intracellular regulators but also as endocrine signaling molecules [106]. miRNAs, encapsulated in exosomes or bound to protein complexes, have been identified in biological fluids like blood, urine and saliva [107]. These circulating miRNAs demonstrate remarkable stability and can be transported to distant tissues, where they exert regulatory effects on target cells. In the context of metabolic regulation, adipose tissue‐derived miR‐34a has been found to act as an adipokine, influencing hepatic lipid metabolism [108, 109], and miR‐375 has been shown to influence insulin sensitivity in peripheral tissues [110]. The stability and tissue‐specific expression patterns of circulating miRNAs make them promising biomarkers for metabolic disorders [111]. Numerous high‐impact studies have extensively characterized the miRNome associated with various metabolic conditions [56, 106, 112, 113]. Here, we highlight only miRNAs relevant for the most diffuse and common metabolic disorders. Circulating levels of miR‐122, miR‐140‐5p, and miR‐142‐3p have been associated with BMI and waist circumference [114], while miR‐126, miR‐146a and miR‐29a have shown potential in predicting the development of Type 2 Diabetes (T2D) [115, 116]. Serum levels of miR‐122, miR‐34a, and miR‐192 have been correlated with the severity of non‐alcoholic fatty liver disease (NAFLD) [117], suggesting their detection as a potential non‐invasive diagnostic tools. In NAFLD, aberrant expression of miR‐34a and miR‐122 exacerbates both lipid accumulation and insulin resistance in hepatocytes. The resulting hepatic steatosis and impaired glucose metabolism create a vicious cycle that can progress to more severe liver disease. In this context, modulation of specific miRNAs has shown promise in preclinical models for simultaneously addressing dyslipidemia and glucose intolerance. For instance, inhibition of miR‐33 was shown to increase HDL levels while also improving insulin sensitivity [86]. Similarly, restoration of miR‐122 levels in models of NAFLD was shown to reduce hepatic steatosis and improve glucose tolerance [117]. While the potential of extracellular miRNAs as biomarkers is promising, several challenges remain. Standardization of miRNA isolation and quantification methods for the development of reliable diagnostic tests is necessary. Integration of miRNA data with other molecular and clinical parameters may enhance the predictive power of these biomarkers. The dual role of miRNAs as endocrine signaling molecules and potential biomarkers opens new avenues for understanding and managing metabolic disorders [106]. Continued research in this field may unveil innovative diagnostic and therapeutic approaches for metabolic diseases.

**Table 1:** Summary of key miRNAs involved in glucose and lipid metabolism. The table shows the main miRNAs, their target genes, their associated functions or effects, and the primary metabolic processes they influence.

microRNA(s)	Target(s)	Function/effect	Metabolic process
miR‐7	Cytoskeletal proteins, ERLIN2	Regulation of insulin release/Indirect activation of SREBP1	Glucose/Lipid metabolism
miR‐9	INS, Onecut2, Granuphilin/Slp4	Modulation of insulin expression and insulin exocytosis	Glucose metabolism
miR‐21	HMGCR	Regulation of cholesterol biosynthesis	Lipid metabolism
miR‐27a	PPARγ	Regulation of adipogenesis	Lipid metabolism
miR‐33a/b	Enzymes in the mitochondrial respiratory chain, SIRT6, ABCA1, CROT, CPT1A, HADHB	Regulation of ATP generation and insulin secretion/Regulates cholesterol efflux and fatty acid oxidation	Glucose/Lipid metabolism
miR‐34a	LDHA, APOB	Modulation of mitochondrial pyruvate oxidation/Affects LDL particle assembly	Glucose/Lipid metabolism
miR‐122	PK, SOCS3, SREBP1	Regulation of pyruvate generation/Indirect activation of SREBP1	Glucose/Lipid metabolism
miR‐124a	Rab27A, Noc2	Modulation of insulin granule docking and fusion	Glucose metabolism
miR‐125a, miR‐143	HK2	Regulation of glycolysis	Glucose metabolism
miR‐128, miR‐148a	LDLR	Modulation of LDL uptake	Lipid Metabolism
miR‐130a/b, miR‐152	GCK, PDHA1	Modulation of ATP/ADP ratio, insulin synthesis and secretion	Glucose metabolism
miR‐135	PFK	Regulation of aerobic glycolysis	Glucose metabolism
miR‐143	GLUT1	Regulation of glucose uptake and glycolysis	Glucose metabolism
miR‐185	PCSK9	Regulation of LDLR expression	Lipid metabolism
miR‐206	GCK	Affects glucose sensing in β‐cells	Glucose metabolism
miR‐375	PDK1, MTPN	Regulation of insulin secretion and granule fusion	Glucose metabolism
miR‐613	LXRα	Regulation of cholesterol efflux and fatty acid synthesis	Lipid metabolism

### miRNAs and the immune response

The immune system is a complex network of cells, tissues, and organs defending the body against pathogens like bacteria, viruses, fungi, and parasites, as well as abnormal cells such as cancer cells. We can imagine the immune system as a sophisticated security network protecting a vast metropolis. In this defense mechanism, two main divisions exist: the innate immune system, our body's rapid response team, and the adaptive immune system, our specialized forces that learn and remember. In particular, the adaptive immune system offers a more targeted and specific response, developing over time as it encounters and remembers specific pathogens. This branch involves B lymphocytes, which produce antibodies for humoral immunity, and T lymphocytes, responsible for cell‐mediated immunity. Moreover the immune system's ability to distinguish between self and non‐self is fundamental, as dysregulation can lead to autoimmune disorders. The relevance of the adaptive immune system in medicine is demonstrated by the concepts of immunoediting [118] and by the success of immunotherapy in cancer treatment [119].

### miRNAs as regulators of B‐cell development and function

B‐cell development occurs in the bone marrow and lymphoid organs, progressing through distinct stages from progenitor cells to mature B cells (naive, memory, and plasma cells). This process involves critical events like V(D)J recombination and class‐switch recombination, which are essential for antibody production and immune responses. miRNAs play critical roles throughout this developmental process [120]. The essential nature of miRNAs in B‐cell development is underscored by studies showing that genetic deletion of Dicer, a key enzyme in miRNA processing, results in a developmental block at the pro‐B‐ to pre‐B‐cell transition [121, 122]. This finding emphasizes the role of miRNAs in immune cell development and opens up questions about the evolutionary significance of these small RNAs in shaping the immune system.

Gene rearrangements in B‐cell receptor (BCR) loci enable B cells to express a specific BCR on their cell surface, a defining characteristic of B‐cell development [123]. During early B cell development miR‐34a inhibits the expression of Foxp1, a transcription factor essential for B cell development [124], while the miR‐17 ~ 92 cluster contributes to B cell development [125, 126]. Recent evidence using conditional mutagenesis has revealed important nuances in its function. While complete knockout of miR‐17 ~ 92 severely impairs B cell development, specific disruption of just the miR‐17 ~ 92/Bim interaction has a surprisingly limited impact [127]. This indicates that although Bim was previously considered a critical target, it represents only one component in a broader network of functionally relevant targets. The miR‐17 ~ 92/Bim axis appears to function more as a molecular ‘rheostat’ that fine‐tunes cell survival in specific contexts, rather than serving as the predominant mechanism driving B cell development. Also, the miR‐15 family is a critical regulator of B cell development, particularly in controlling progenitor B cell expansion and the transition from proliferation to differentiation. The control of B cell development is regulated by two key mechanisms: direct suppression of cell cycle regulators (Cyclins E1, D3, and Cdc25a) and repression of IL‐7 receptor expression. Loss of the miR‐15 family leads to excessive proliferation and defective differentiation of pre‐B cells, envisaging this miRNA family as a molecular switch, which is regulated by both IL‐7R and pre‐BCR signaling to balance proliferation and differentiation during B cell development [128, 129].

Shifting the attention to mature B cells and their activation, miR‐155 emerges as a central player, promoting germinal center responses and regulating class‐switch recombination. By targeting Activation‐Induced Cytidine Deaminase (AID), miR‐155 tunes antibody diversity and specificity [130, 131]. However, miR‐155′s role extends beyond these functions. It is indeed rapidly upregulated during the activation of naive B cells, playing an essential role in promoting B cell proliferation and survival through the repression of targets such as PU.1 and SHIP‐1. SHIP‐1, an inhibitor of TLR/PI3K/Akt kinase pathways, is particularly important as its downregulation enhances B‐cell responsiveness to activating signals [132]. Concurrently, miR‐146a is also upregulated downstream of NF‐κB signaling, where it also functions as a negative regulator [133]. This miRNA‐dependent B cell regulation provides a mechanism to modulate B cell receptor signaling intensity and prevent excessive activation. In addition, while miR‐181b drives the differentiation of B cells without altering myeloid cells or T cells [131], miR‐150 regulates pro‐B to pre‐B transition, controls B1 cell development and inhibits plasma cell differentiation by targeting c‐Myb, a TF relevant for lymphocyte development [134]. The miR‐212/miR‐132 cluster is induced upon B cell activation and upregulated in germinal center B cells. Their main targets are FOXO3, involved in cell survival and proliferation, and BCL6, a key regulator of germinal center formation [122]. miR‐212/132 also regulate FOXP1 and SOX4 expression in activated B cells, modulating B cell activation dynamics, germinal center reactions and memory B cell function [122, 135]. On the contrary, the miR‐30 family facilitates plasma cell differentiation by targeting Blimp‐1 [136]. miRNAs also play crucial roles in regulating developmental switches, as evidenced by the Let‐7–Lin28b–Arid3a regulatory axis [137, 138]. This axis controls the divergence between B1 cells, that are produced in the fetus and self‐renew in the periphery, and conventional B2 cells, which are produced postnatally and replenished in the bone marrow. Lin28b, which is highly expressed in fetal liver B cell progenitors, inhibits let‐7 miRNA maturation. Consequently, let‐7 miRNAs are more abundant in adult bone marrow B cell progenitors, where they target Arid3a, a key transcription factor. This intricate interplay acts as a developmental switch, determining B1 versus B2 cell fate. Finally, in mature B cells, different miRNAs regulate antibody and cytokine production through various mechanisms: (a) miR‐185 influences B cell activation and antibody production by targeting Bruton's Tyrosine Kinase (BTK), a key component of B cell receptor signaling, regulating in this way the strength of B cell activation signals; (b) miR‐21 promotes plasma cell differentiation and antibody secretion by targeting Pten and Pdcd4, in turn enhancing the Akt/mTOR pathway and facilitating the metabolic changes necessary for high‐level antibody production in plasma cells; (c) miR‐126 regulates plasma cell differentiation by targeting the transcription factor IRF4, relevant for immunoglobulin secretion; (d) miR‐146a acts as a negative regulator of TLR signaling in B cells, preventing excessive inflammatory responses and autoimmune reactions [139]. Collectively, we illustrated how miRNAs form a sophisticated regulatory network that calibrates B‐cell responses, ensuring appropriate antibody production, affinity maturation, and memory formation while maintaining immune homeostasis and preventing excessive or misdirected reactions. Looking forward, the potential for miRNAs to influence B cell memory and long‐lived plasma cells, which are essential for long‐term immunity, is intriguing. Understanding these processes could have profound implications for vaccine development and for the treatment of antibody‐mediated diseases.

### miRNAs as regulators of T‐cell development, differentiation and immune functions

T‐cell development is an intricate, multistage process that occurs within the thymic microenvironment [140]. This process begins with the migration of hematopoietic progenitors from the bone marrow to the thymus, guided by chemokine gradients [141, 142]. Starting as double‐negative cells (CD4^−^ and CD8^−^), they undergo TCR gene rearrangement and progress to double‐positive cells (CD4^+^ and CD8^+^). Through selection processes that test their functionality and eliminate self‐reactive cells, they mature into single‐positive CD4^+^ or CD8^+^ T cells before entering circulation as naive T cells [143]. Mature single‐positive T cells upregulate the S1P1 receptor, enabling their egress from the thymus into the periphery as naive T cells [144].

Throughout the complex process of differentiation, essential transcription factors, such as Notch1, GATA3, Bcl11b, and specific signaling pathways guide T cell fate and development, inducing the generation of a diverse T cell repertoire capable of recognizing a wide range of foreign antigens while maintaining tolerance to self‐antigens, thus forming the basis of effective adaptive immunity [145]. For clarity and comprehensive understanding, we will dissect our discussion of miRNA involvement in T cell biology into two distinct yet interconnected phases: T‐cell development and mature T cell function. This approach will highlight the dynamic nature of miRNA regulation throughout the T‐cell lifecycle. It is important to note that some miRNAs actually play roles in both developmental and mature T cell contexts. By examining miRNA regulation in these two phases, we explore the critical role of miRNA in T cell biology, revealing cell‐specific expression patterns that influence development and effector function [146, 147, 148]. As reported in Table 2, the miR‐17 ~ 92 cluster is essential for the transition from double‐negative (DN, CD4^−^ and CD8^−^) to double‐positive (DP, CD4^+^ and CD8^+^) thymocytes. This cluster has been proposed to function through multiple mechanisms, including the repression of the pro‐apoptotic protein BIM [149, 150] and regulation of IL‐7 receptor expression [151]. Studies using compound genetic deletion models have suggested a potential role for BIM in this process [151]. However, it is important to note that such genetic approaches, while informative, do not necessarily establish direct regulatory relationships. Additional mechanistic studies using approaches that specifically disrupt miRNA binding sites would be valuable to definitively determine the relative contribution of BIM and other targets to miR‐17 ~ 92‐mediated T‐cell development.

**Table 2:** miRNAs in T‐cell development. The table shows the main miRNAs, their target genes, their associated developmental stages or cell types, and their primary functions during T cell maturation.

microRNA(s)	Direct/indirect target(s)	Function/effect	Stage/cell type
miR‐17 ~ 92 cluster	Bim, PTEN	Promotion of survival and proliferation	DN to DP transition
miR‐146a	TRAF6, IRAK1	Modulates TCR signaling strength and T cell tolerance	Late development
miR‐150	c‐Myb	Regulation of early T‐cell development	Maturing thymocytes
miR‐155	SOCS1	Regulation of Treg development in the thymus	Thymic Treg
miR‐181a	DUSP5, DUSP6	Rheostat of TCR signaling sensitivity	DP thymocytes

Similarly, miR‐142 also plays a crucial role in T‐cell development, influencing thymocyte proliferation. While miR‐142 targets Cdkn1b, a cell cycle inhibitor, this interaction alone does not fully account for its effects on thymocyte proliferation. Loss of miR‐142 leads to reduced thymocyte proliferation and accumulation of early thymic progenitors, suggesting a complex regulatory network involving multiple target genes that collectively contribute to miR‐142's function in T‐cell development. [152]. In DP thymocytes, miR‐181a is highly expressed and functions as a rheostat for TCR signaling. Specifically, it enhances TCR signaling sensitivity during positive and negative selection processes by targeting phosphatases, including DUSP5 and DUSP6 [153]. In contrast with miR‐181a, miR‐150 regulates early T‐cell development by targeting c‐Myb, with its expression progressively increasing as thymocytes mature [142, 154]. Of particular note is miR‐155, which exhibits a dual function in T cell subsets: it is involved in thymic regulatory T cell (Treg) development, by targeting SOCS1, and in Th17 cell differentiation by suppressing ETS1, a negative regulator of Th17 lineage commitment [155]. Finally, in the later stages of development, miR‐146a emerges as a critical modulator of TCR signaling strength and T cell tolerance. It achieves this by targeting two key signaling molecules: TRAF6 and IRAK1, in turn tuning the T‐cell response towards antigen stimulation [156, 157].

Once CD4^+^ and CD8^+^ lineages are committed, a diverse repertoire of naive T cells capable of recognizing a wide range of antigens is produced. T cell differentiation starts when naive T cells encounter their cognate antigens in secondary lymphoid organs. This process transforms naive T cells into specialized effector or memory T cells capable of mounting targeted immune responses. CD4^+^ T cells can differentiate into various subsets, including T helper 1 (Th1), T helper 2 (Th2), T helper 17 (Th17), T follicular helper (Tfh), and Tregs, each with distinct functions and cytokine profiles [140, 158]. CD8^+^ T cells primarily differentiate into cytotoxic T lymphocytes (CTLs) and memory cells [159, 160]. The differentiation pathway is determined by the strength of T cell receptor (TCR) engagement, co‐stimulatory molecules, and the cytokine milieu present during activation. Key transcription factors, such as T‐bet for Th1, GATA3 for Th2, RORγt for Th17, and Foxp3 for Tregs, drive the expression of subset‐specific genes and give plasticity for functional adaptation of immune environments [140, 161]. In Table 3, we summarize the main miRNAs involved in T‐cell development and function. In naive CD4^+^ T cells, miR‐125b suppresses genes involved in T cell differentiation, including IFNG, IL2RB, IL10RA, and PRDM1. This suppression aligns with synthetic miR‐125b interventions, which inhibits differentiation to effector cells [162]. It remains highly expressed in naive and memory T cell populations but decreases in effector T cells. Conversely, miR‐142‐3p is inhibited by FoxP3, increasing cAMP production and enhancing Treg cell suppressor function. [163]. A key component in T cell functions is miR‐21. It is rapidly upregulated upon TCR stimulation and promotes T cell survival by targeting pro‐apoptotic genes like PDCD4 [160, 164, 165]. miR‐21 is particularly appealing because it influences the balance between Tregs and Th17 cells by modulating the expression of SMAD7, a negative regulator of TGF‐β signaling, and also by indirectly enhancing FoxP3 expression [166, 167]. The Th17/Treg dichotomy represents a critical balance in the immune system between pro‐inflammatory and regulatory responses [168]. This balance is crucial for immune homeostasis, autoimmune disease prevention and cancer immunity, as we will discuss later. Multiple miRNAs orchestrate Treg development and function through distinct mechanisms. While miR‐31 directly represses FoxP3 by binding its 3′ UTR, miR‐21 indirectly enhances its expression [167].

**Table 3:** miRNAs in T cell differentiation and function. The table shows the main miRNAs, their target genes, their associated developmental stages or cell types, and their primary functions during T cell maturation.

microRNA(s)	Direct/indirect target(s)	Function/effect	Stage/cell type
miR‐17, miR‐19b	TGF‐β signaling components	Th1 and Tfh cell differentiation	Th1, Tfh cells
miR‐21	PDCD4, SMAD7	Regulation of T cell survival and Treg/Th17 balance	Activated T cells, Tregs
miR‐24, miR‐27	IL‐4	Th2 cell function	Th2 cells
miR‐31	FoxP3	Regulation of FoxP3 expression and suppressive capacity	Tregs
miR‐92a, miR‐125a	EOMES, IL‐10R	Tr1 differentiation	Tr1 cells
miR‐125b	IFNG, IL2RB, IL10RA, PRDM1	Inhibits differentiation to effector cells	Naive CD4+ T cells
miR‐142‐3p	GARP	Regulation of T cell activation and proliferation	Naive and memory T cells
miR‐155	ETS1/CTLA4	Th17 differentiation/Treg differentiation	Th17
miR‐182	FOXO1	T cell proliferation and survival	Activated T cells
miR‐326	Ets‐1	Th17 differentiation	Th17 cells

The miR‐15/16 family plays an essential role in maintaining peripheral tolerance by regulating Treg function and homeostasis. Loss of miR‐15/16 leads to impaired Treg function and altered expression of key Treg proteins, resulting in spontaneous inflammation. Notably, miR‐15/16 prevents excessive proliferation that could shift Treg fate towards an effector phenotype [169]. Another critical regulator is miR‐181a/b‐1, which shows dynamic expression during T‐cell development and is particularly important for thymic Treg generation. It functions by establishing proper TCR signaling thresholds necessary for Treg development. Interestingly, while miR‐181a/b‐1 deficiency impairs thymic Treg generation, it enhances their suppressive capacity in the periphery through post‐transcriptional regulation of CTLA‐4 [170]. Recently, particular interest has been placed on Tr1 cells for their role in maintaining immune tolerance and their potential therapeutic applications in autoimmune diseases and transplantation. Tr1 cells are a subset of regulatory T cells characterized by their high production of IL‐10, lack of FOXP3 expression, and potent capacity to maintain immune tolerance and inflammatory response regulation [171]. miR‐92a and miR‐125a have been identified as potential inhibitors of Tr1 differentiation. These miRNAs target the expression of key Tr1‐associated genes, including Eomesodermin (EOMES) and the Interleukin‐10 Receptor (IL‐10R). By downregulating these genes, miR‐92a and miR‐125a may impede Tr1 cell differentiation and potentially modulate their immunoregulatory capacity [172]. EOMES is also a target of miR‐29, mainly involved in Th differentiation. miR‐29 specifically regulates Th1 differentiation by simultaneously targeting two key transcription factors – T‐bet and EOMES – that promote IFN‐γ production. This dual targeting by miR‐29 serves as a critical brake on the Th1 program, preventing excessive IFN‐γ production and maintaining proper T helper cell balance [173]. These examples demonstrate how different miRNAs can employ distinct strategies—from context‐dependent regulation to multiple target repression—to orchestrate T helper cell differentiation.

Other miRNAs have also been investigated. miR‐326 is notably involved in the differentiation of Th17 cells by targeting Ets‐1 [174, 175], and miR‐155 promotes Th1 and Th17 differentiation by targeting SOCS1 and IFN‐γRα, respectively, also supporting Treg development through SOCS1 inhibition [155]. On the other hand, the same miR‐155 promotes Treg differentiation by suppressing CTLA‐4 [176]. Interest in miR‐155 is increasing because it plays a crucial role in regulating the Treg/Th17 balance, which is a key factor in autoimmune processes, which we will explore in depth in a subsequent section. The dual role of miR‐155 is fascinating, as it exemplifies how a single miRNA can have context‐dependent effects, potentially serving as a molecular switch in T cell fate decisions [177]. Similarly, the miR‐17 ∼ 92 cluster shows versatile functions in T helper cell differentiation. Beyond its established role in promoting Th1 cell differentiation through targeting TGF‐β signaling components [178, 179, 180], it is also critical for Tfh development and function. In Tfh cells, miR‐17 ∼ 92 functions as a master regulator of cell identity through two mechanisms: promoting the expression of Tfh‐specific genes and repressing non‐Tfh gene programs, including direct suppression of Rorα. This dual regulation ensures proper Tfh cell function in supporting B cell‐mediated antibody responses [177]. Other miRNAs govern T cell differentiation and function. miR‐24 and miR‐27 have a role in inhibiting Th2 cell function in a coordinated fashion, via inhibition of IL‐4 production [120]. miR‐182 instead plays a significant role in the context of T cell activation and clonal expansion. Its expression is induced by STAT5 signaling, which is activated downstream of TCR stimulation and IL‐2 engagement. Upon induction, miR‐182 targets FOXO1 and, by doing so, miR‐182 helps to release the brake on T cell expansion and effector function. This regulatory axis (STAT5 → miR‐182 ⊣ FOXO1) forms a feedforward loop that promotes T cell proliferation and survival during immune responses [181].

Specific to CD8^+^ T cells, miR‐150 was shown to regulate cells' cytolytic activity [182], while miR‐139 and miR‐342 were shown to modulate the expression of effector molecules by targeting EOMES and Ezh2, respectively [183]. miR‐181 plays a crucial role in CD8^+^ T cell responses during viral infections; specifically, it impairs the generation of antigen‐specific CD8^+^ effector T cells [184]. In the context of memory formation, miR‐15/16 influences memory CD8^+^ T cell differentiation by targeting Bcl2 and Ccne [185], a process complementing memory T‐cell development in CD4^+^ lineages. Interestingly, some miRNAs that regulate CD4^+^ T cell subsets also play roles in CD8^+^ T cell function. An interesting example is miR‐21, which we discussed as influencing the Treg/Th17 balance but which also promotes CD8^+^ T cell survival by targeting pro‐apoptotic genes and which is more highly expressed in effector and memory CD8^+^ T cells compared to naive cells [186]. This dual functionality underscores the versatility of miRNAs in regulating multiple aspects of T cell biology. The phenomenon of T cell exhaustion, particularly relevant in chronic infections, is also regulated by miRNAs in CD8^+^ T cells. miR‐31 promotes CD8^+^ T cell exhaustion in chronic infections [187], while miR‐155 helps maintain CD8^+^ T cell responses in these conditions [188], adding another layer in T cell biology. miR‐181 exemplifies how miRNA dysregulation can impact immunity across the lifespan: its age‐related decline particularly affects CD8+ T cell responses, leading to delayed viral clearance [184] and potentially contributing to the increased susceptibility to infections observed in elderly individuals.

These miRNAs collectively contribute to the complex regulatory network governing T‐cell development, activation, and differentiation, highlighting the multifaceted roles of miRNAs in shaping T‐cell‐mediated immunity. The examples that we have highlighted underscore a broader question: to what extent does the overlap in miRNA function between metabolism and immunity reflect an evolutionary strategy for coordinated cellular responses? Finally, are all these miRNAs acting only cell‐autonomously or is their function associated with specific secretion by active T cells, as shown for Tregs [189]? The potential for miRNA‐mediated intercellular communication within the immune system represents a frontier in research. If broadly validated, this concept could transform our understanding of immune regulation and open new avenues for immunotherapy.

### miRNAs in immunometabolism

**Fig. 2:**
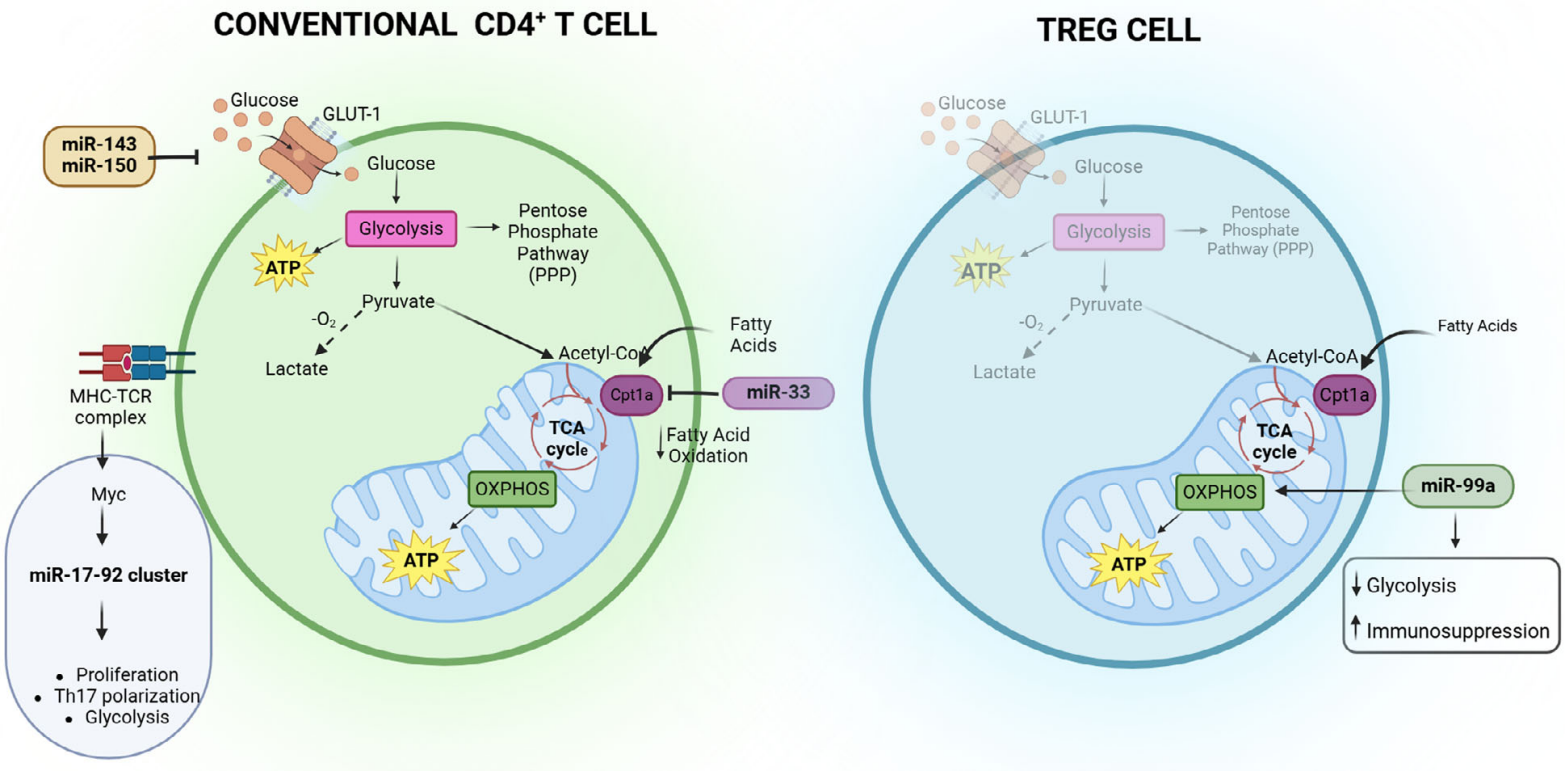
Immunometabolism regulation of miRNAs in CD4^+^ T cells. Representation of the miRNAs involved in regulating the metabolic pathways of conventional CD4^+^ T cells and CD4^+^ Treg cells. Left: Conventional CD4^+^ T cells show typical metabolic features regulated by miRNAs. miR‐143 and miR‐150 act as negative regulators of glucose metabolism by suppressing GLUT1 expression, thereby limiting glucose uptake in the cell, while miR‐33 functions as an inhibitor of fatty acid oxidation by targeting Cpt1a. The miR‐17‐92 cluster, which is induced by Myc‐mediated TCR signaling, promotes instead thymocyte proliferation, regulating Th17 polarization and glycolytic enhancement. Right: Metabolic regulation of miRNAs in CD4^+^ Tregs. miR‐99a plays a central role by enhancing oxidative metabolism while suppressing glycolysis. This metabolic reconfiguration augments their immunosuppressive capacity.

Immune metabolism is a growing field that explores the interplay between cellular metabolism and immune function [190]. Metabolic reprogramming is not merely a consequence of immune cell activation but an essential driver of immune responses: this is a paradigm shift in immunology. Naive T cells can remain quiescent for years until they are activated by antigen stimulation on the T cell receptor (TCR). Antigen stimulation leads to their transition to effector T cells and memory T cells. Naive and memory T cells predominantly rely on oxidative phosphorylation while TCR‐activated and effector T cells primarily rely on glycolysis [191]. This glycolytic switch, mediated by the upregulation of glucose transporter GLUT1 and glycolytic enzymes, supports rapid proliferation and effector function [192]. After activation, on a per cell basis, there is about a fivefold expansion of both the translational and metabolic machineries; the metabolic flux increases 8‐fold, with glycolysis increasing more than respiration (65‐fold vs 4‐fold) [193]. In addition, activated T cells are divided into conventional and regulatory subsets; the first is responsible for the immune response, the second is fundamental in blunting an excessive immune response [194]. Tregs demonstrate metabolic plasticity, utilizing both glycolysis and fatty acid oxidation, with a preference for lipid metabolism in stable Tregs [195, 196]. However, the metabolic regulation of immune responses is further complicated by the influence of the tissue microenvironment [197]. Nutrient availability, oxygen tension, and the presence of metabolites can significantly modulate immune cell function. For instance, tumor microenvironments often create metabolically restrictive conditions that impair T cell effector functions, contributing to immune evasion. It is evident that miRNAs serve as critical modulators of these metabolic shifts. One issue is whether miRNAs affect immunometabolism by targeting directly metabolic enzymes or indirectly through preferential targeting of transcription factors that have a pleiotropic role. A detailed review has recently described the numerous miRNAs that are directly involved in immunometabolism, and these include miR‐143 and miR‐150, which inhibit Glut1 expression and consequent glucose entry into the cell, and miR‐33, which inhibits synthesis of Cpt1a, an enzyme that promotes fatty acid oxidation, as shown in Fig. 2, as well as the AMPK pathway [198]. However, given their pleiotropic role, most miRNAs act through multiple targets and mechanisms. For example, the miR‐17 ~ 92 cluster has been shown to play a multifaceted role in T cell metabolism, with miR‐19b enhancing glycolysis to support Th17 differentiation, and miR‐17 modulating fatty acid metabolism to favor Tfh cell development [199]. Interestingly, miR‐17 ∼ 92 expression is induced by Myc‐mediated TCR signaling and promotes thymocyte proliferation by suppressing the translation of Pten [149], thus explaining Myc's mechanistic impact on Th17 polarization and glycolysis. MiR‐99a expression is induced by transforming growth factor β (TGF‐β) [200], the classic pathway associated with Treg formation [201], and *in vivo* it enforces Treg oxidative metabolism, impairs glycolysis, and increases immune suppression [202] (Fig. 2). Likewise, let‐7 can potentially target mTOR and cause differentiation of T cells and can also markedly affect the metabolic switch of stimulated CD8^+^ T cells by targeting Myc [203, 204].

Furthermore, the metabolism of dendritic cells (DCs) is regulated by miRNAs. Specifically, miR‐142 plays a crucial role in the metabolic reprogramming of DCs, enabling the switch from oxidative phosphorylation (OXPHOS) to glycolysis. This metabolic shift is necessary for DCs to mount an effective immunogenic response. In the absence of miR‐142, DCs fail to undergo this metabolic transition, resulting in reduced production of pro‐inflammatory cytokines and impaired T cell activation [192, 205]. Additionally, miR‐5119 has been identified as a potential regulator of PD‐L1 in DCs. Interestingly, mimic‐engineered DC vaccines incorporating miR‐5119 have shown promising results in enhancing anti‐tumor immune responses in a mouse model of breast cancer [206]. Conversely, pathogens can exploit miRNA‐mediated regulation of DCs to their advantage. A study has shown that *Helicobacter pylori* can downregulate miR‐375, leading to the inhibition of DC maturation and contributing to the development of gastric cancer [207]. Intriguingly, recent research has revealed that microRNA‐mediated metabolic reprogramming is also an important strategy employed by *Mycobacterium tuberculosis* (Mtb) to evade host immune responses. Mtb infection was shown to upregulate the expression of miR‐144‐3p in macrophages [208]. Specifically, in this scenario, by targeting peroxisome proliferator‐activated receptor α (PPARα) and ATP‐binding cassette transporter A1 (ABCA1), miR‐144‐3p was shown to promote lipid accumulation and bacterial survival. Accordingly, miR‐144‐3p inhibition was shown to have the opposite effects. Mtb infection was also shown to downmodulate miR‐26a levels, leading to the upregulation of its main target, the histone deacetylase SIRT6. Upregulated SIRT6 then suppresses glycolysis, inhibits expression of HIF‐1α‐dependent glycolytic genes, regulates intracellular succinate levels, and controls the release of pro‐inflammatory cytokines [209].

**Fig. 3:**
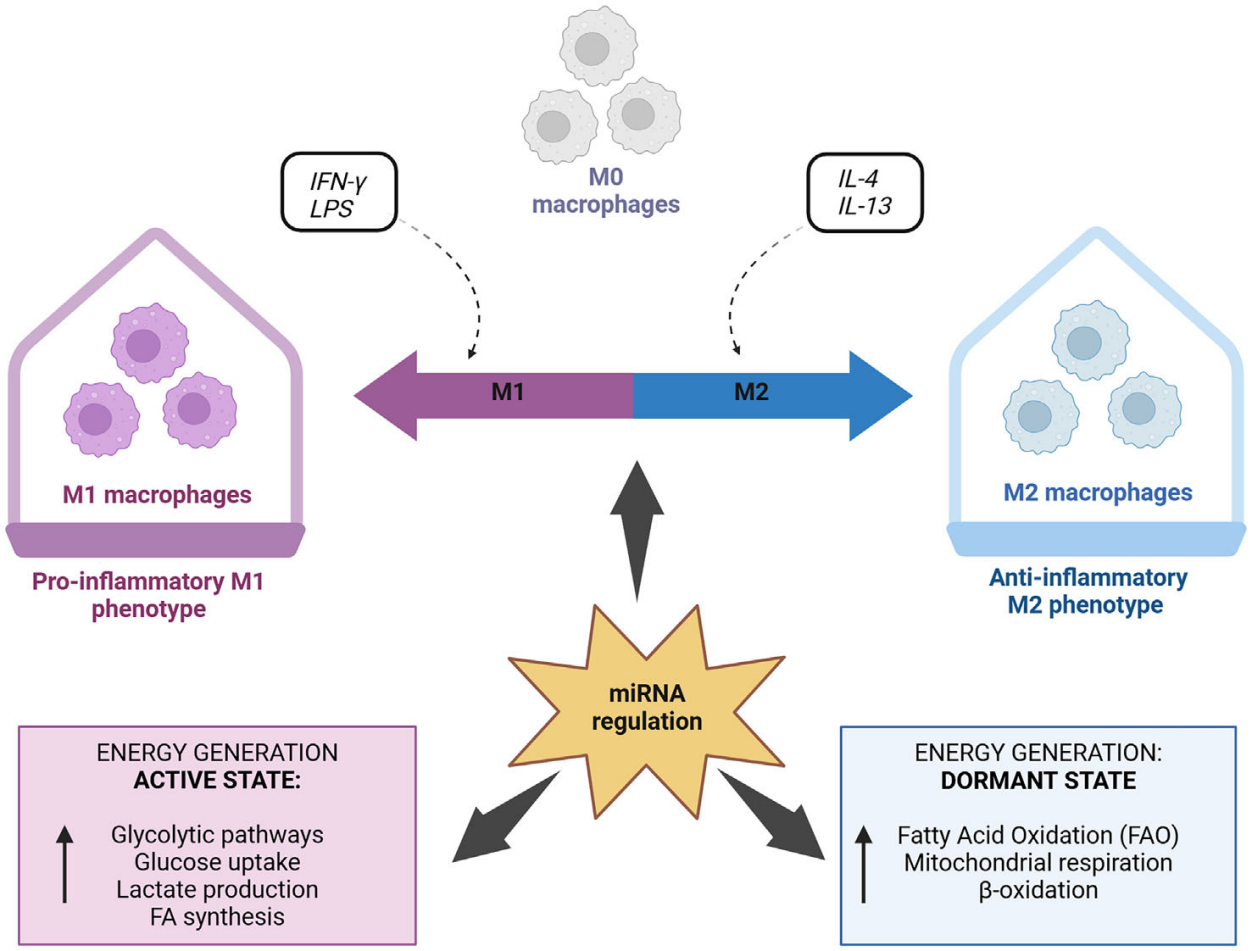
miRNA‐mediated regulation of macrophage metabolic reprogramming. A simplified representation of the complex interplay between the pro‐inflammatory M1 and anti‐inflammatory M2 macrophage phenotypes, and the critical role of miRNAs in controlling their metabolic states. IFN‐γ and LPS are the major stimulators of M1 polarization, whereas IL‐4 and IL‐13 are inducers of M2 polarization. On the left, the M1 macrophage exhibits an “Active State” metabolism characterized by glycolytic pathways, glucose uptake, lactate production, and fatty acid synthesis. In contrast, the M2 macrophage on the right demonstrates a “Dormant State” metabolism, relying more on fatty acid oxidation (FAO), mitochondrial respiration, and β‐oxidation. In the center, the “miRNA regulation” icon highlights the critical regulatory function of miRNAs in modulating the M1‐M2 macrophage transition and in controlling their metabolic states. Please refer to the text for more details.

miRNAs regulate macrophage metabolism by targeting key metabolic regulators like AMPK, PI3K, and mTOR. During M1 polarization, metabolic reprogramming shifts from oxidative phosphorylation to glycolysis, supporting increased energy demands for antigen presentation and pathogen response [192]. Indeed, M1 macrophages utilize aerobic glycolysis for rapid energy production during pro‐inflammatory responses, contrasting with M2 macrophages that rely on fatty acid oxidation (FAO) (Fig. 3). miR‐33 plays a crucial role in regulating macrophage inflammatory polarization by targeting AMPK, balancing mitochondrial oxidative phosphorylation and aerobic glycolysis [210]. Accordingly, *Mycobacterium tuberculosis*‐induced miR‐33 inhibits mitochondrial FAO, causing lipid accumulation in macrophages by targeting the AMPK pathway. miR‐223 prevents macrophage lipid accumulation and atherosclerosis by activating the PI3K pathway [211] while miR‐141/200c regulates macrophage polarization by modulating AMPK and mTOR signaling, with its depletion associated with M2 phenotype shifts [212].

Unfortunately, many other cases describing the impact of miRNA on immunometabolism remain more difficult to interpret mechanistically given the lack of knowledge of specific mRNA targets. This limitation represents both a challenge and an opportunity for future research. Developing more sophisticated tools to identify and validate miRNA targets in specific cellular contexts could advance our understanding of immunometabolism. Finally, it is conceivable that several miRNAs that affect the metabolism of lymphocytes are derived from adjacent cells. It should not be forgotten that more than 90% of T cells are resident within tissues [213] and by interacting with antigen presenting cells, can endocytically engulf foreign miRNAs. Indeed, circulating exosomes might control CD4^+^ T cell immunometabolic functions via the transfer of miRNAs such as miR‐142 [214]. Intercellular miRNA transfer in immunometabolism suggests that immune regulation extends beyond cell‐autonomous mechanisms. The plasticity of immunometabolism, in short, may strongly rely on the capability of miRNAs to fine‐tune gene expression.

### miRNA dysregulation in immune cells: Focusing on the Treg/Th17 balance in autoimmune diseases

miRNA dysregulation in cells, particularly in immune cells, can have profound implications in health and disease, contributing to a wide array of pathological conditions. This dysregulation can manifest as either upregulation or downregulation of specific miRNAs, leading to aberrant cellular functions and immune responses. The involvement of miRNAs in specific diseases has been extensively described during the last years. As a result, a selected number of miRNAs have been assessed in preclinical studies and clinical trials. Examples include: MRG‐229 and MRG‐201, two miR‐29 mimics—the first is at the preclinical stage and was developed and tested as a treatment for pulmonary fibrosis [215], while the second completed clinical trials in 2021 and was developed for keloid and fibrous scar formation treatment [216]; mimics of miR‐466c, which by increasing VEGFA expression are being explored as potential treatments for peripheral artery disease and heart failure; MRG‐110, an inhibitor of miR‐92a, which completed a phase 1 clinical trial in 2019, showing safety and efficacy in humans [217]; and RGLS8429, an inhibitor of miR‐17, which has completed a phase 1 clinical trial for autosomal dominant polycystic kidney disease [216, 218]. These are only a few of the miRNA applications that are currently being explored, highlighting the importance of basic research for the discovery of novel miRNA targets [219, 220].

As the interplay between miRNAs and immune function in disease is intriguing, here we will discuss and focus on a specific example: the Treg/Th17 balance and the consequences of its dysregulation in autoimmune disease. As we mentioned above, a new perspective on autoimmunity centers on the Treg/Th17 balance [168, 221], evidencing the delicate equilibrium required for proper immune function. Maintaining the Treg/Th17 balance is crucial for developing effective autoimmune disease treatment strategies. Treg cells and Th17 cells are two CD4^+^ T lymphocyte subsets with opposing actions: Treg cells are essential for immunological tolerance and for suppression of excessive immune responses [222, 223]; Th17 cells promote inflammation and protect against extracellular pathogens [163, 224, 225]. How can dysregulation of this balance lead to various pathological conditions? (1) An increase in Th17 cells and/or a decrease in Treg function leads to autoimmune disease; (2) insufficient Treg suppression or overactive Th17 responses result in chronic inflammation; and (3) increased Treg localization in tumors can suppress anti‐tumor immunity, while Th17 cells can have both pro‐ and anti‐tumor effects depending on the microenvironment. miRNAs have emerged as critical regulators in Treg/Th17 homeostasis. Some miRNAs involved in both Treg and Th17 cell differentiation and function have been described in the previous paragraph.

**Fig. 4:**
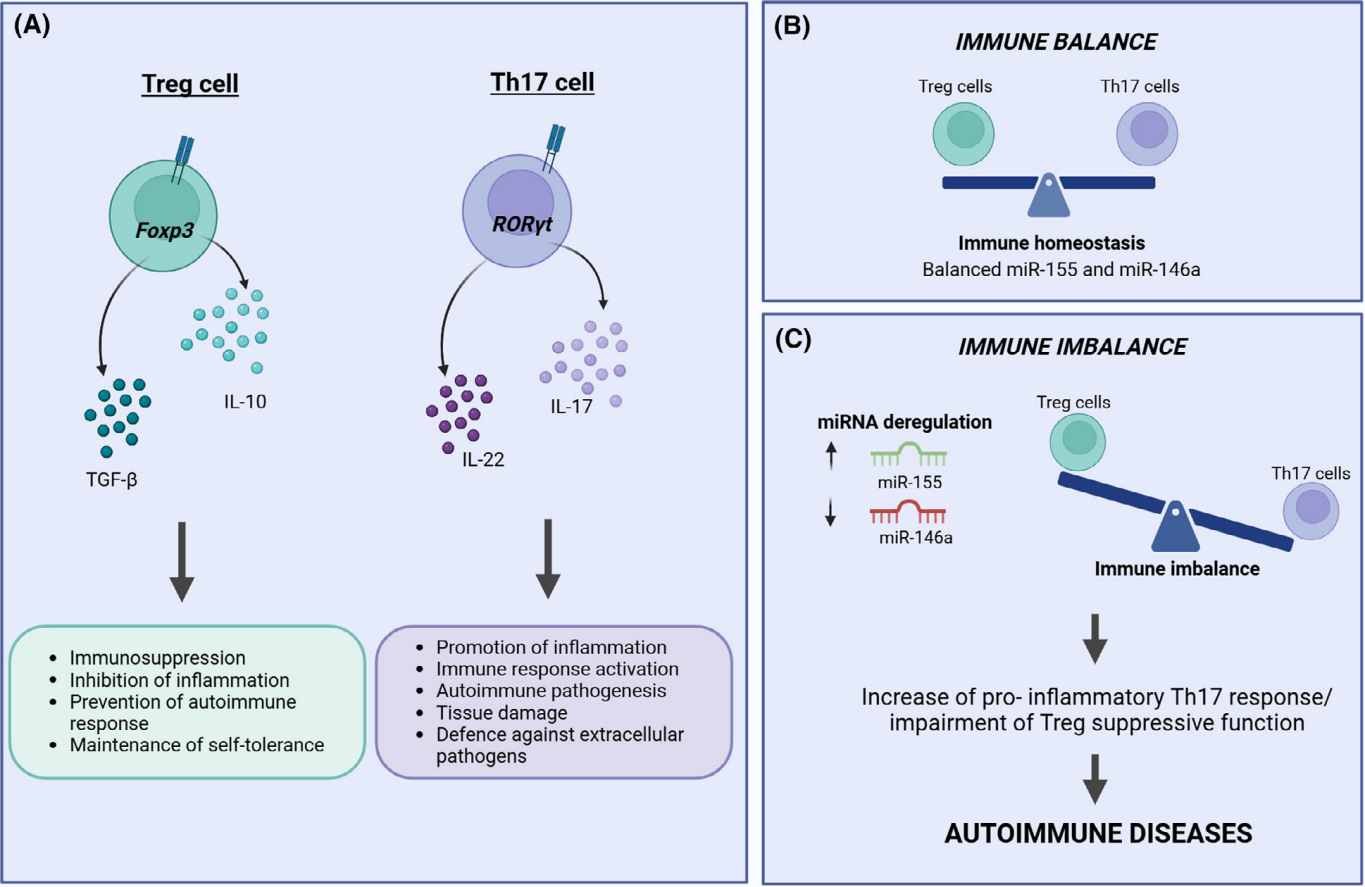
miRNA‐mediated regulation of Treg/Th17 balance. A simplified representation of the role of miRNAs in modulating the balance between Tregs and Th17 cells in immune regulation. (A) Functions of Tregs and Th17 cells. Tregs, which are characterized by the transcription factor Foxp3, secrete the immunosuppressive cytokines IL‐10 and TGF‐β, promoting immunosuppression, inhibiting inflammation, preventing autoimmune responses and maintaining self‐tolerance. Conversely, Th17 cells, defined by the transcription factor RORγt, produce the pro‐inflammatory cytokines IL‐17 and IL‐22, leading to inflammation, activation of immune responses, autoimmune pathogenesis, and tissue damage. (B) Immune balance state in which Treg and Th17 cells are in an equilibrium, maintained by fine‐tuned expression of miR‐155 and miR‐146a. This balance is crucial for appropriate immune responses and self‐tolerance. (C) An immune imbalance scenario, associated with autoimmune diseases. Here, miRNA dysregulation—more specifically upregulation of miR‐155 and concomitant downregulation of miR‐146a –disrupt the Treg/Th17 ratio, resulting in increased pro‐inflammatory Th17 responses and impaired Treg suppressive function, ultimately contributing to the development of autoimmune diseases.

Here, we will specifically focus on miR‐155/miR‐146a. miR‐155 has a crucial role in T cell and Treg differentiation. Specifically, miR‐155 knockout mice showed impaired immune responses and a bias towards Th2 differentiation [226]. miR‐155 was shown to be involved in regulating Treg cell differentiation, maintenance, and function by targeting FoxP3 [227, 228]. Through a feedback loop, FoxP3, in turn, binds to the miR‐155 precursor sequence, maintaining high miR‐155 expression in Tregs [228]. miR‐155 maintains IL‐2R signaling by targeting SOCS1, a negative regulator of the IL‐2 pathway. This inhibition of SOCS1 promotes STAT5 phosphorylation, enhancing Treg proliferation and survival. However, the role of SOCS1 in Treg function is complex and not fully defined, as SOCS1 knockout in Tregs leads to loss of FoxP3 expression, suggesting context‐dependent effects of the miR‐155‐SOCS1 axis in Treg biology [229]. miR‐155 also acts as a positive regulator of Th17 cell differentiation and function, but through mechanisms distinct from those in Tregs. While miR‐155 enhances STAT3 signaling in Th17 cells, SOCS1 targeting is dispensable for miR‐155‐mediated Th17 differentiation [229, 230], suggesting that miR‐155 regulates Th17 cells through alternative targets. Recent studies have shown that in autoimmune conditions such as multiple sclerosis, miR‐155 is often overexpressed [231], leading to an imbalance in the Treg/Th17 ratio. This dysregulation not only enhances pro‐inflammatory Th17 responses but also impairs Treg function, despite its positive role in Treg development (Fig. 4). This cell type‐specific effect of miR‐155 highlights the context‐dependent nature of miRNA function and the delicate balance required for immune homeostasis. miR‐146a is instead crucial specifically for Treg cell function and immune response modulation. Its deficiency increases the number of Tregs while impairing their immunosuppressive capacity [227]. Predominantly expressed in Tregs, miR‐146a regulates their ability to suppress Th1 responses by targeting STAT1, a key transcription factor in Th1 differentiation and IFN‐γ signaling. This regulation parallels SOCS1‐mediated STAT1 inhibition, thereby enhancing the overall effect [232]. miR‐146a dysregulation typically results in enhanced inflammatory responses. For example, reduced expression of miR‐146a leads to overactivation of its target pathways, particularly NF‐κB signaling, contributing to chronic inflammation and autoimmune pathogenesis [227]. These findings open up exciting possibilities for therapeutic interventions. Targeted manipulation of miR‐155 and/or miR‐146a levels in the context of autoimmune diseases could potentially help reestablish the Treg/Th17 equilibrium and reduce symptoms. However, miRNAs are just one piece of the puzzle in Treg/Th17 regulation. Several factors contribute to its complexity: (1) cell plasticity—Th17 and Treg cells can interconvert and so the balance is not just about cell numbers, but also about the stability of cell phenotypes; (2) the influence of the microenvironment, since local tissue factors significantly impact cell differentiation and function; (3) temporal dynamics—the balance shifts during immune responses and disease progression; and (4) cellular metabolism, which influences T cell fate and function. In conclusion, while miRNAs are important regulators, they operate within a broader network of factors, including TFs, cytokines, and metabolic pathways. Therefore, a comprehensive understanding of the Th17/Treg balance requires an integrated approach that considers multiple regulatory elements beyond miRNAs, emphasizing the need for different strategies in studying and targeting this critical aspect of immune regulation.

## miRNAs in the era of artificial intelligence (AI) and machine learning (ML)

Traditional research methods, while valuable, have limitations in fully capturing the multifaceted nature of miRNA networks and their systemic effects. Recent studies have demonstrated the context‐dependent nature of miRNA interactions, highlighting the need for more sophisticated analytical approaches [233, 234]. For example, a recent comprehensive study on miR‐181 in developing murine T cells revealed the nuanced nature of miRNA targeting mechanisms [165], evidencing how miR‐181 is not confined to a single regulatory strategy, but acts predominantly through RNA destabilization, utilizing different and alternative seeds.

Similarly, miR‐155 was also shown to function differentially depending on the specific immune cell type analyzed [235]. These studies highlight the need for more sophisticated analytical approaches that can capture the intricate and dynamic nature of miRNA‐mediated gene regulation. Clearly, miRNA interactions are far more complex than what was previously thought, involving multiple regulatory mechanisms that depend on specific cellular contexts.

How can we effectively harness the vast complexity of miRNA regulation? How can we gain deeper insights and develop novel therapeutic strategies? These open questions led to a paradigm shift in miRNA research, with the integration of Artificial Intelligence (AI) and Machine Learning (ML) techniques. The transition to AI‐driven approaches in miRNA biology is a necessary evolution to address the data generated by genomics and transcriptomics. These advanced computational methods offer the potential to uncover patterns, networks and relationships that may be imperceptible to human researchers, potentially unlocking new paradigms in our understanding of gene regulation, disease mechanisms, and therapeutic strategies. The application of AI/ML in miRNA research represents the convergence of biology with computer science and data analysis. Here, we will explore how AI is revolutionizing our understanding and utilization of miRNAs, potentially unlocking new paradigms in diagnostics, therapeutics, and gene regulation. This field of study is still in its infancy, but the landscape is evolving at an unprecedented speed and the prospects are incredibly exciting. AI has emerged as a transformative force in cellular biology, reinventing and modernizing the analysis of complex biological systems. ML algorithms and deep neural networks are now routinely applied to classify cellular phenotypes, predict protein structures, and model gene regulatory networks [236].

Nevertheless, it is critical to consider both the transformative potential and the limitations of these approaches. High‐quality, diverse datasets are essential to train robust AI models. Without reliable and representative input data, even the most sophisticated AI algorithms will inevitably produce meaningless results. Most importantly, the findings from AI‐driven miRNA research must be rigorously validated through direct experimental investigation. Computational predictions cannot replace empirical evidence and mechanistic understanding derived from biological experiments. Failure to bridge the gap between *in silico* and *bench top* can relegate AI‐derived insights to mere theoretical speculation, detached from biological reality.

As we delve into the specific applications of AI in miRNA research, broader AI approaches might be used in integrating diverse biological data types, in order to simulate complex biological systems [237]. In which specific contexts does the use of AI prove to be important in the field of miRNAs? We hypothesize that the main areas that would benefit from such approaches are (1) target prediction and expression and (2) biomarker discovery and miRNA association with disease.

Machine learning models enhance miRNA target prediction by integrating sequence features, evolutionary conservation, and structural characteristics. Deep Learning architectures like convolutional neural networks excel at capturing complex sequence patterns for miRNA‐mRNA interactions. In more detail, ML algorithms, such as support vector machines (SVMs) and random forests, have been employed to integrate diverse features, including sequence complementarity, evolutionary conservation, and structural characteristics of miRNA‐mRNA duplexes [238]. For instance, the DeepMirTar approach utilizes a hierarchical DL framework to predict miRNA targets with high accuracy, outperforming traditional methods [159]. Specifically, by merging sequence information, structural characteristics, and contextual data through sophisticated convolutional and recurrent neural networks, DeepMirTar can detect miRNA‐target interactions with a precision that was unimaginable a decade ago. Besides DeepMirTar, the miTAR tool offers another innovative solution to miRNA target prediction [160]. This tool is based on a hybrid DL approach that integrates convolutional neural networks (CNNs) and recurrent neural networks (RNNs). The unique strength of miTAR lies in its ability to process raw miRNA and gene sequences directly, eliminating the need for extensive pre‐processing or manual feature engineering. By integrating CNNs' spatial feature learning capabilities with RNNs' sequential feature detection, miTAR can uncover intricate molecular interactions that might escape traditional computational methods. This technique not only improves model performance but also helps mitigate overfitting, a persistent challenge in ML applications to biological data. Instead, the miRAW approach introduces a distinctive DL methodology using artificial neural networks (ANN). By designing an ANN capable of processing raw genetic sequences, miRAW eliminates the need for complex pre‐processing steps and enables more nuanced feature extraction [164].

Several platforms using ML in miRNA target prediction showcase the power of ensemble ML approaches. Utilizing a complex interplay of support vector machines, random forest algorithms, and gradient boosting techniques, these tools do not simply predict; they synthesize. They weave together experimental validation with predictive computational models, creating an enrichment of molecular understanding. Machine learning methods detect both canonical (seed complementarity) and non‐canonical miRNA‐mRNA interaction sites by incorporating numerous features representing sequence, structure, conservation, and molecular context [238]. Examples include: ChimiRic [165] and miRTarget [235] which employ SVMs for binary classification of potential interactions, and TarPmiR [239], which uses random forest methodology to estimate the probability of a target site's authenticity.

More importantly, AI algorithms can analyze complex patterns in miRNA expression profiles across various tissue types and disease stages, identifying specific miRNA combinations that serve as robust biomarkers. For instance, in cancer research, AI‐driven analyses have revealed miRNA signatures that can distinguish between tumor subtypes, predict metastasis risk, and even indicate therapy response [240]. Recent studies across different cancer types illustrate the profound potential of ML in biomarker discovery. In gastric cancer research, ML algorithms, including SVMs, identified a panel of 29 miRNAs with remarkable diagnostic accuracy. The study revealed critical miRNAs associated with mortality and early detection potential [241]. Similarly, in colorectal cancer, metabolomics combined with ML techniques identified key metabolite biomarkers. SVMs demonstrated exceptional discriminatory power, highlighting the potential of advanced computational approaches in disease diagnostics [242]. Another compelling example comes from esophageal squamous cell carcinoma (ESCC) research, where a sophisticated five‐miRNA signature was developed to predict overall survival. Using advanced techniques like Cox regression, recursive feature elimination, and LASSO regression, researchers created a risk score model that could effectively stratify patient prognosis [243]. The AI models are trained on datasets containing miRNA expression profiles from various cancer types and stages, along with corresponding clinical data. During the training process, the algorithms learn to identify patterns and combinations of miRNA expressions that are most informative for specific diagnostic or prognostic tasks. Once trained, these models can be applied to new, unseen data for predictions [244]. Considering the extensive potential outlined here, AI models can effectively predict novel connections between miRNAs and diseases, guiding experimental research and suggesting potential therapeutic interventions. These models integrate diverse data types, including miRNA expression profiles, known disease associations, and functional genomics data, to infer new miRNA‐disease relationships. AI‐driven predictions have led to the discovery of previously unknown roles of miRNAs in various pathologies, from neurodegenerative disorders to metabolic diseases. Furthermore, these predictions help prioritize miRNAs for experimental validation, accelerating the process of translating miRNA research into clinical applications. Last but not least, AI‐driven analysis also helps in elucidating complex miRNA‐mediated regulatory networks, providing insights into the broader impact of miRNAs on cellular processes. Advanced ML techniques, such as graph neural networks, can model the intricate interactions between miRNAs, their targets, and other regulatory elements [245]. This approach allows researchers to identify key nodes in regulatory networks and predict the systemic impact of miRNA perturbations. Such analyses are particularly valuable in understanding the role of miRNAs in complex biological processes like development, immune responses, and disease progression. The predictive power of these AI systems extends beyond pure academic curiosity. In the realm of precision medicine, these computational frameworks offer a myriad of possibilities. By analyzing miRNA expression patterns across diverse disease states, ML models can potentially identify molecular signatures that precede clinical manifestations. However, this technological promise is not without profound challenges. The very power of these computational methods introduces complex questions. How do we interpret the “black box” of advanced neural networks? How can we ensure that our computational models capture biological reality rather than generating mathematically elegant but biologically spurious connections? We must maintain scientific humility. Every computational model remains an approximation of biological complexity. Even the most sophisticated AI approach needs careful experimental validations. Our computational models must be viewed as hypotheses to be rigorously tested, not as definitive biological truths. Perhaps the most critical advancement is not technological, but human. We now require researchers who are equally fluent in ML algorithms and molecular biology—individuals capable of translating between computational abstractions and biological mechanisms.

## Limitations of this review

The miRNA field has exploded in few years. Starting from the first paper published in early 2000's, it rapidly reached 1000 articles/year in 2007, and 10 000/year in 2014. This review aims at a generalist description of this field, with all the contradictions that such an explosive growth can generate. We apologize for the omissions and hope that we have been able to convey an idea of the complexity of the field and its potential evolution.

## Conclusions and perspectives

This comprehensive review has tried to illuminate the landscape of miRNA biology, focusing on the multifaceted roles of miRNAs in metabolism, immune function, and the emerging field of immunometabolism. Moving forward, several critical areas warrant further exploration to fully leverage the potential of miRNAs in biomedical research and clinical practice: first, exploring miRNA‐mediated regulation in immunometabolism and second, developing precise miRNA‐based immunotherapies. As we look to the future, the integration of AI and ML approaches in miRNA research opens new avenues for discovery. These computational tools promise to enhance our ability to predict miRNA targets and uncover novel biomarkers. However, challenges remain, particularly in data integration, model interpretability, and translating *in silico* predictions to biological problems. As we stand at this scientific frontier, the marriage of miRNA research and AI represents more than a technological evolution. We are not merely developing new tools; we are expanding biological knowledge, creating a more nuanced, dynamic understanding of the complex molecular systems. Each computational breakthrough is a step towards an advanced comprehension of genetic regulation. The future of miRNA research is not about AI replacing human insight, but about creating a powerful symbiosis—a collaborative intelligence that amplifies our ability to decode the fundamental complexity of life.

## Conflict of interest

The authors declare no conflicts of interest.

## Author contributions

SO planned and wrote the original manuscript. NM and SB revised the manuscript.
